# Performance of a Web-Based Reference Database With Natural Language Searching Capabilities: Usability Evaluation of DynaMed and Micromedex With Watson

**DOI:** 10.2196/43960

**Published:** 2023-04-17

**Authors:** Angela Rui, Pamela M Garabedian, Marlika Marceau, Ania Syrowatka, Lynn A Volk, Heba H Edrees, Diane L Seger, Mary G Amato, Jacob Cambre, Sevan Dulgarian, Lisa P Newmark, Karen C Nanji, Petra Schultz, Gretchen Purcell Jackson, Ronen Rozenblum, David W Bates

**Affiliations:** 1 Division of General Internal Medicine Brigham and Women's Hospital Boston, MA United States; 2 Clinical and Quality Analysis Mass General Brigham Somerville, MA United States; 3 Harvard Medical School Boston, MA United States; 4 Massachusetts College of Pharmacy and Health Sciences (MCPHS) Boston, MA United States; 5 Department of Anesthesia, Critical Care and Pain Medicine Massachusetts General Hospital Boston, MA United States; 6 Merative Cambridge, MA United States; 7 Vanderbilt University Medical Center Nashville, TN United States; 8 Intuitive Surgical Sunnyvale, CA United States; 9 Harvard TH Chan School of Public Health Boston, MA United States

**Keywords:** medication safety, patient safety, usability, searching behavior, efficiency, quality of care, web-based databases, point-of-care information, POCI, point-of-care tools, artificial intelligence, machine learning, clinical decision support, natural language processing

## Abstract

**Background:**

Evidence-based point-of-care information (POCI) tools can facilitate patient safety and care by helping clinicians to answer disease state and drug information questions in less time and with less effort. However, these tools may also be visually challenging to navigate or lack the comprehensiveness needed to sufficiently address a medical issue.

**Objective:**

This study aimed to collect clinicians’ feedback and directly observe their use of the combined POCI tool DynaMed and Micromedex with Watson, now known as DynaMedex. EBSCO partnered with IBM Watson Health, now known as Merative, to develop the combined tool as a resource for clinicians. We aimed to identify areas for refinement based on participant feedback and examine participant perceptions to inform further development.

**Methods:**

Participants (N=43) within varying clinical roles and specialties were recruited from Brigham and Women’s Hospital and Massachusetts General Hospital in Boston, Massachusetts, United States, between August 10, 2021, and December 16, 2021, to take part in usability sessions aimed at evaluating the efficiency and effectiveness of, as well as satisfaction with, the DynaMed and Micromedex with Watson tool. Usability testing methods, including think aloud and observations of user behavior, were used to identify challenges regarding the combined tool. Data collection included measurements of time on task; task ease; satisfaction with the answer; posttest feedback on likes, dislikes, and perceived reliability of the tool; and interest in recommending the tool to a colleague.

**Results:**

On a 7-point Likert scale, pharmacists rated ease (mean 5.98, SD 1.38) and satisfaction (mean 6.31, SD 1.34) with the combined POCI tool higher than the physicians, nurse practitioner, and physician’s assistants (ease: mean 5.57, SD 1.64, and satisfaction: mean 5.82, SD 1.60). Pharmacists spent longer (mean 2 minutes, 26 seconds, SD 1 minute, 41 seconds) on average finding an answer to their question than the physicians, nurse practitioner, and physician’s assistants (mean 1 minute, 40 seconds, SD 1 minute, 23 seconds).

**Conclusions:**

Overall, the tool performed well, but this usability evaluation identified multiple opportunities for improvement that would help inexperienced users.

## Introduction

### Background

Answering health care providers’ drug and disease questions in an accurate, effective, and efficient manner can be challenging. Common information-seeking issues that providers face include struggling to navigate through large amounts of information, being unaware of particular wording needed for optimal general search results, and requiring excessive time and effort to find answers [1-3]. Solutions may include the use of point-of-care information (POCI) tools, such as web-based databases that use evidence-based information to aid clinicians with drug and disease questions [4]. Commonly used drug and disease information systems that are considered POCI tools include UpToDate, DynaMed, Micromedex, and BMJ Best Practice [4,5]. POCI tools increase a provider’s ability to answer clinical questions in a timely manner, which can improve overall patient safety and care [4]. However, difficulties with searching in a manner that leads to a satisfactory answer may occur for various reasons, including the user not knowing when to stop searching or being unaware of how the POCI tool prefers clinical questions to be asked [6]. Artificial intelligence (AI) can be used to enhance POCI tools and has been integrated with electronic health records and clinical decision support systems to assist providers in improving patient and drug safety [7]. By combining AI capabilities, such as natural language processing (NLP), with the comprehensiveness of a POCI tool, there is potential to quickly answer a clinician’s questions and reduce mental fatigue compared with a manual search [8]. NLP is used in a variety of applications in health care and has been shown to assist in more efficient retrieval of information [9-11].

The DynaMed and Micromedex with Watson combined solution, now known as DynaMedex, is a POCI tool that includes drug and disease information with AI capabilities for information retrieval [12,13]. EBSCO partnered with IBM Watson Health, now known as Merative, to develop the combined tool, which aims to assist clinicians with answering clinical questions using evidence-based information [14,15]. This system combines the existing tools DynaMed, Micromedex, and Watson Assistant into an all-in-one web platform [16]. DynaMed is a medical condition knowledge database that contains summaries of evidence-based research, guidelines, clinical photographs, and other additional resources [17]. DynaMed provides peer-reviewed clinical content for 28 specialties on disease topics, health conditions, abnormal findings, disease evaluation, differential diagnosis, and disease management [16]. Micromedex is a pharmacological knowledge base with supporting literature curated for clinical significance by experts [18]. Micromedex is one of the largest web-based reference databases for medication information and provides detailed information on drug-drug interactions, drug monographs, and management of drug reactions [16,18]. Micromedex is often used by health systems to support the clinician in medication therapy management and patient education [8]. The purpose of combining DynaMed with Micromedex was to bring together drug and disease content into a single source that could be used to aid clinicians in making informed clinical decisions [12]. Watson Assistant is an AI-based conversational agent powered by IBM’s DeepQA supercomputer Watson, which aids users in information retrieval through a combination of NLP and machine learning [19]. Other research and applications of NLP in health care today focus on pulling important information from patient records to aid in decision-making, whereas Watson Assistant is a conversational agent that responds to user questions [9,11,20] related to drug information, drug interactions, and intravenous compatibility by mining databases of evidence-based information [8,16]. Drug information topics include drug classes, dosing, administration, medication safety, mechanism of action, and pharmacokinetics [7]. A prior study demonstrated Watson Assistant’s potential to answer clinician questions; a reported 80% of queries within Watson Assistant’s domain of knowledge were correctly classified by the conversational agent [7]. The paper also provides detailed information about the system architecture of Micromedex, including Watson Assistant.

### Objectives

The objective of this study was to collect clinicians’ feedback and directly observe their use of the combined tool to identify potential areas for improvement and assess participants’ perceptions to inform further development. Specifically, we focused on whether provider roles made a difference in their experience of using DynaMed and Micromedex with Watson. Little research exists on the user interaction and usability of these types of tools for health care providers. We evaluated the usability of the combined tool to determine how well users were able to reach their search goals with efficiency, effectiveness, and satisfaction. We asked participants to test the combined tool by using both the general search function and Watson Assistant throughout the testing session to evaluate benefits and challenges arising from using either feature to search for information. From these findings, we summarized key themes that were observed or raised by providers in varying roles. In doing so, we generated recommendations for improving the clinician experience while using the tool.

## Methods

### Overview

This summative usability study collected data on how participants used DynaMed and Micromedex with Watson to complete information-searching tasks for a set of clinical scenarios. We report on quantitative usability metrics as well as describe observed differences in user experience among roles and experience with reference tools [21-23]. IBM provided a free subscription to the tool to conduct usability testing.

### Ethics Approval

This research project was reviewed and approved by the Mass General Brigham institutional review board (2021P000139).

### Informed Consent

Verbal informed consent was obtained from all individual participants included in the study. As part of recruitment, participants were informed in writing that their deidentified data from the audio and video recordings would be used for research.

### Recruitment

Clinicians were recruited from inpatient and outpatient sites affiliated with 2 academic medical centers in Boston, Massachusetts, United States: Brigham and Women’s Hospital and Massachusetts General Hospital. From August 10, 2021, to December 16, 2021, recruitment emails were sent to physicians, pharmacists, registered nurses (RNs), nurse practitioners (NPs), and physician’s assistants (PAs) practicing in the following specialties: internal medicine, neurology, cardiology, oncology or hematology, infectious diseases, and endocrinology. The participant population was chosen based on the intended users of the tool. To achieve a sufficient sample size across clinicians in each role, general care RNs and pharmacists were also recruited. Clinicians were recruited using purposive and network sampling strategies. Participants were compensated for participation.

Before testing, participants were asked about their clinical role, years spent in practice, whether they practiced in an outpatient or inpatient setting, and whether they had prior experience using DynaMed and Micromedex with Watson. Participants were assigned a participant ID that was used on all study and data collection materials [21].

### Scenario and Script Development

The study’s research pharmacists (HHE, DLS, and MGA) compiled a list of real-world questions supplied by clinical pharmacists from various specialty areas for both inpatient and outpatient settings. These questions were then evaluated and categorized by specialty and clinical area. The pharmacists determined whether each question could be answered accurately by DynaMed and Micromedex with Watson using the *general search* function or *Watson Assistant*. A set of questions that could be answered were selected and reworded into clinical scenarios for usability testing (Multimedia Appendix 1). Scenario question content was developed to be relevant to common situations according to specialty. Each usability session included 7 scenarios that covered a range of different clinical areas (Table 1).

**Table 1:** Assigned question categories for each script. The scripts included 7 scenarios covering these question categories.

Question categories	Clinical specialty scripts	Nursing script	Pharmacy script
Adverse drug reaction or toxicity	✓	✓	✓
Disease	✓		
Drugs of choice or indication or therapeutic	✓		✓
Dosing or kinetics	✓		✓
Interaction (drug or herb or laboratory or disease)	✓	✓	✓
Monitoring or laboratory test	✓	✓	✓
Pregnancy or lactation or breastfeeding	✓	✓	✓
Drug administration		✓^a^	
Stability or compatibility		✓	✓

^a^Registered nurses received 2 drug administration questions: either an inpatient or outpatient drug administration question depending on their primary work setting and a second drug administration question regardless of setting.

A total of 56 scenarios were created for usability testing. Eight unique scripts were created: cardiology, endocrinology, hematology or oncology, infectious diseases, internal medicine, neurology, nursing, and general pharmacy (Multimedia Appendix 1). For all scripts, the following question categories required the user to initiate their search using the Watson Assistant functionality: adverse drug reaction or toxicity, interaction (drug or herb or laboratory or disease), and pregnancy or lactation. All other question categories required the clinician to use the general search functionality to find the answer for the scenario. Physicians, the NP, and PAs were assigned a script based on their specialty. Pharmacists were either assigned a general pharmacy script or a specialty script to have sufficient sample sizes for each script type. RNs were assigned a nursing script.

### Pilot Testing

A pilot usability test was conducted to refine the testing procedure. The participating clinician was given a version of the internal medicine usability script that contained the scenarios and posttask questions. Scenarios that were confusing to the participant were reviewed and reworded to avoid misinterpretation.

### Usability Testing Procedure

**Figure 1:**
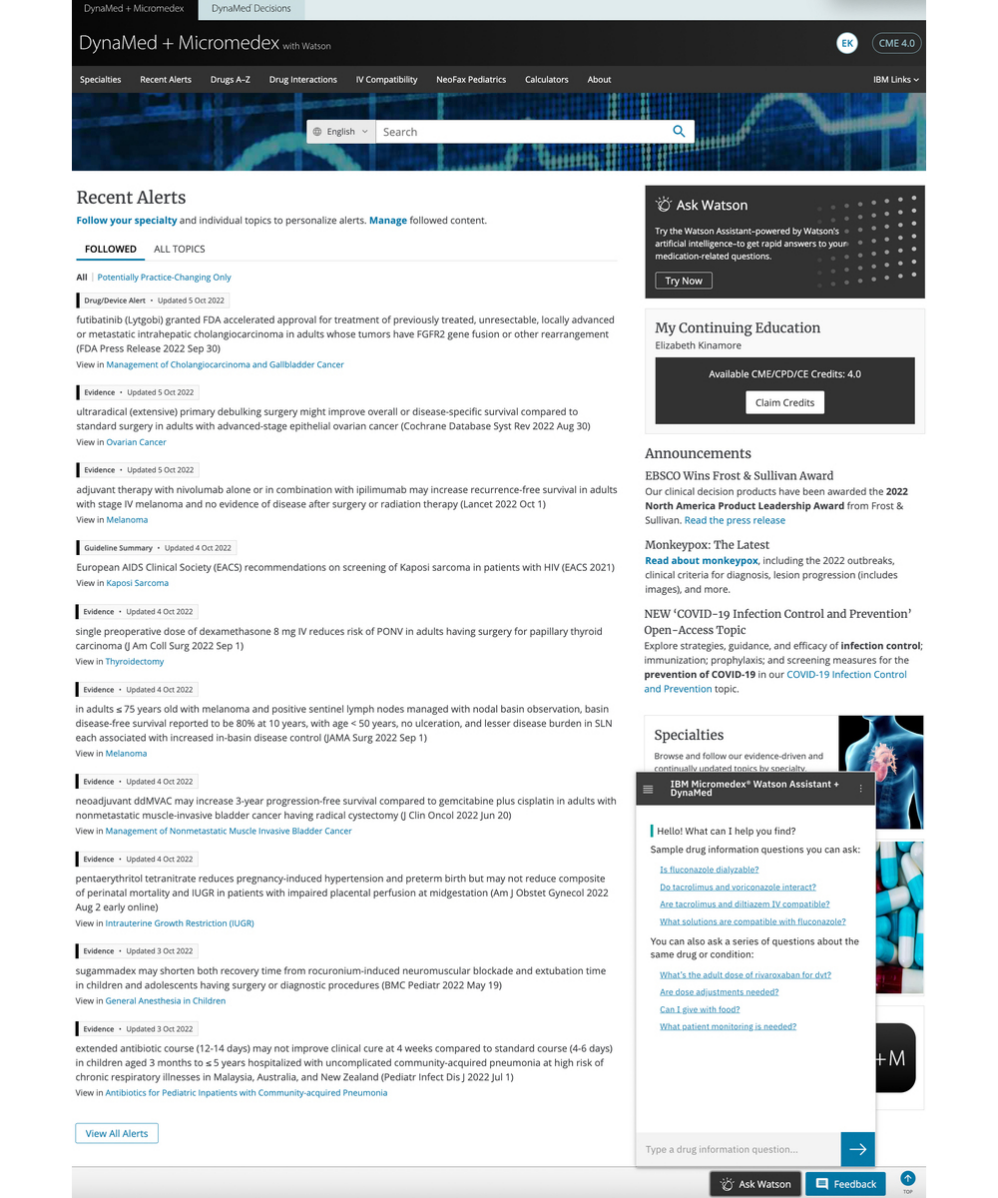
Screenshot of DynaMed and Micromedex with Watson home page. Features include the general search bar and Watson Assistant. Since the time of the study, the user interface has been updated slightly (image courtesy of Merative, used with permission).

Each usability session was conducted remotely (ie, via Zoom [Zoom Video Communications, Inc]) to address both safety and scheduling concerns amid the COVID-19 pandemic. Participants were informed of the nature of the study as well as the scenario testing procedure and given the opportunity to ask questions related to testing. They were also informed of the moderator’s role as a neutral observer and the research assistant’s role in recording data. Verbal informed consent was obtained to record the audio and video of the Zoom session. Participants were asked a series of demographic questions and about their experience with reference databases for disease and medication management (Multimedia Appendix 1). Next, using the chat function in Zoom, the moderator (PMG) sent the participants the web address to access the DynaMed and Micromedex with Watson tool. Participants were asked to open the web page and begin screen sharing. The moderator provided no training on the tool but did ensure that the participants knew where to locate the general search function and Watson Assistant (Figure 1). Next, the moderator asked the participant to read each scenario aloud and search for the answer using either the general search function or Watson Assistant as detailed in the task. As the participant used the tool, they were encouraged to verbalize their thought processes, expectations for specific functionality, and reactions to elements in the tool. If the participant was able to find the answer, they informed the moderator of the answer and that they had completed the task. The participant could end the task at any time.

In situations where the participant encountered an unexpected usability issue that prevented them from moving forward with the current task or subsequent tasks, the moderator provided a prompt that assisted the participant in discovering why they were encountering the issue. These assists were not intended to help participants navigate content but rather were provided after multiple unsuccessful attempts to use a specific tool feature that was preventing them from accessing content; for example, assists were provided to participants who were not able to move forward with a task because they were unaware that it was necessary to clear filters on Watson Assistant at the beginning of each search to ensure that the conversational agent incorporated the correct keywords when searching for information.

When the participant finished the task, they were asked to respond to 2 posttask questions, administered through the polling feature in Zoom. The first posttask question required the participant to rate the ease of finding the answer on a 7-point Likert scale ranging from 1=*very difficult* to 7=*very easy*. The next posttask question asked the participant to rate their level of satisfaction with the answer using a 7-point Likert scale ranging from 1=*very dissatisfied* to 7=*very satisfied*. The participants were asked to explain their reasoning for each score. Finally, a semistructured posttest interview was conducted with the participants (Multimedia Appendix 1). Participants were able to provide their likes, dislikes, recommendations, and other opinions about their experience using the tool. The answers to the posttest interview questions were transcribed by the research assistant as the participant answered the questions.

### Analysis

During the usability testing sessions, data were logged into an Excel (Microsoft Windows 7; Microsoft Corp) spreadsheet and organized by participant identification and task number. The recordings of the usability sessions were analyzed by the reviewers (PMG, MM, JC, and SD) who were assigned to observe and record metrics pertaining to task success, time on task, and navigation and search behavior. The start time for each task was marked when the participant finished reading the scenario and asking any clarifying questions. The end time was marked when the participant found an answer or decided to end the task themselves. Technical issues or outside interruptions were removed from the total task time. To analyze navigation and search behavior, we captured the types and number of actions taken by the participants (eg, use of *Find on Page* or Ctrl-F, text entry into the general search function or Watson Assistant dialog box, or clicking on a search result or left navigation menu item). A content analysis was carried out on the posttest interview responses. Similar responses to each question were grouped and counted. All quantitative and qualitative data were compiled into a data set containing all metrics for all participants. The metrics, posttask question scores, and posttest interview answers were then analyzed by clinical question category and 3 role categories (physician, NP, and PA; pharmacist; and RN). We grouped the physicians, the solitary NP, and the PAs into 1 role category because they all work directly with patients to diagnose and treat health conditions. Descriptive statistics are reported for all quantitative metrics.

## Results

### Participant Demographics

Usability sessions were completed with 43 participants who had been practicing for an average of 10 years (Table 2). Of the 14 pharmacists, 5 (36%) received a specialty script (n=3, 60% had a specialty in cardiology and were assigned a cardiology script; n=1, 20% specialized in infectious diseases; and n=1, 20% specialized in hematology or oncology), and the remaining 9 (64%) received a general pharmacy script. Of the 2 RNs, 1 (50%) practiced in an inpatient setting, and 1 (50%) practiced in an outpatient setting. Of the remaining 27 clinicians, 21 (78%) were physicians, 1 (4%) was an NP, and 5 (19%) were PAs, and their specialties included cardiology (n=2, 7%), endocrinology (n=6, 22%), internal medicine (n=6, 22%), infectious disease (n=4, 15%), hematology or oncology (n=4, 15%), and neurology (n=5, 19%).

Most of the pharmacists (12/14, 86%) reported using Micromedex daily or at least once a week, whereas the physicians, NP, and PAs reported using Micromedex less than once a week in their current practice (22/27, 82%). Of the 21 physicians, 1 (5%) reported daily use of the combined solution on their mobile phone (Table 3).

**Table 2:** Participant demographics (N=43).

Characteristics				Participants, n (%)
**Clinical role**				
	Physician			21 (49)
	Nurse practitioner			1 (2)
	Physician’s assistant			5 (12)
	Pharmacist			14 (33)
	Registered nurse			2 (5)
**Specialty used to assign script**				
	**Cardiology**			
		Physician	2 (5)	
		Pharmacist	3 (7)	
	**Endocrinology**			
		Physician	5 (12)	
		Nurse practitioner	1 (2)	
	**Internal medicine**			
		Physician	5 (12)	
		Physician’s assistant	1 (2)	
	**Infectious diseases**			
		Physician	4 (9)	
		Pharmacist	1 (2)	
	**Hematology or oncology**			
		Physician	2 (5)	
		Physician’s assistant	2 (5)	
		Pharmacist	1 (2)	
	**Neurology**			
		Physician	3 (7)	
		Physician’s assistant	2 (5)	
	Nursing: registered nurse			2 (5)
	General pharmacy: pharmacist			9 (21)
**Years in practice**				
	<5			18 (42)
	5-9			9 (21)
	10-14			6 (14)
	15-19			5 (12)
	≥20			5 (12)
**Hospital setting**				
	Outpatient			15 (35)
	Inpatient			20 (47)
	Both outpatient and inpatient			8 (19)

**Table 3:** Reported frequency of Micromedex use (N=43).

	Daily, n (%)	Once a week, n (%)	Once a month, n (%)	Few times a year, n (%)	Never, n (%)
Physicians, nurse practitioner, and physician’s assistants (n=27)	2 (7)	3 (11)	3 (11)	5 (19)	14 (52)
Pharmacists (n=14)	6 (43)	6 (43)	N/A^a^	N/A	2 (14)
Registered nurses (n=2)	N/A	N/A	1 (50)	N/A	1 (50)
Total	8 (19)	9 (21)	4 (9)	5 (12)	17 (40)

^a^N/A: not applicable.

### Ease of Finding and Satisfaction With the Answer

All participants (N=43) completed the 7 scenarios for a total of 301 tasks. A participant was unable to use Watson Assistant because there were technical issues; therefore, they completed all 7 scenarios with the general search function only. The overall average ease of finding an answer was 5.68 (SD 1.57) out of 7 (physicians, NP, and PAs: 5.57, SD 1.64; pharmacists: 5.98, SD 1.38; and RNs: 5.07, SD 1.69). In 71% (5/7) of the clinical question categories, the pharmacists rated the ease of finding the answer higher than the physicians, NP, and PAs (Table 4). The largest difference between pharmacist ratings of ease of finding the answer (mean 6.36, SD 1.34) and physician, NP, and PA ratings (mean 5.19, SD 1.92) was in the adverse drug reaction or toxicity category (Table 4).

Overall, average satisfaction with the answer was 5.97 (SD 1.54) out of 7 (physicians, NP, and PAs: 5.82, SD 1.6; pharmacists: 6.31, SD 1.34; and RNs: 5.57, SD 1.79; Table 5). The pharmacists gave a rating of >6 to all but 2 question categories (disease and dosing or kinetics). The average ratings by the physicians, NP, and PAs ranged from 5.33 (SD 1.69) for the disease category to 6.19 (SD 1.21) for the drugs of choice or indication or therapeutic questions. As with average ease, the question category with the largest difference in satisfaction rating between the physicians, NP, and PAs (average 5.89, SD 1.67) and the pharmacists (average 6.71, SD 0.83) was adverse drug reaction or toxicity (Table 5).

Responses to overall ease and satisfaction varied by specialty (Table 6). The infectious disease specialists (pharmacists: 1/14, 7%, and physicians: 4/21, 19%) rated the ease of finding an answer as 4.89 (SD 1.62) out of 7 in comparison with the cardiology specialists, who had the highest average ease rating (6.11, SD 1.51). Average satisfaction with an answer was rated the highest by the pharmacists (6.44, SD 1.24) using the general pharmacy script compared with the internal medicine specialists, who rated satisfaction with the answer as 5.31 (SD 1.88).

**Table 4:** Average ease of finding the answer by question category and role.

Question category	Tasks, n	Overall average (SD)	Physicians, nurse practitioner, and physician’s assistants (n=27), average (SD)	Pharmacists (n=14), average (SD)	Registered nurses (n=2), average (SD)
Adverse drug reaction or toxicity^a^	43	5.56 (1.79)	5.19 (1.92)	6.36 (1.34)	5.00 (1.41)
Disease	32	5.66 (1.26)	5.56 (1.31)	6.2 (0.84)^b^	N/A^c^
Drugs of choice or indication or therapeutic	41	5.63 (1.69)	5.70 (1.46)	5.50 (1.51)	N/A
Dosing or kinetics	41	5.15 (1.90)	4.89 (1.99)	5.64 (1.69)	N/A
Drug administration	4	6.25 (2.92)	N/A	N/A	6.25 (0.96)
Interaction^a^	43	5.65 (1.77)	5.81 (1.78)	5.79 (1.37)	2.50 (2.12)
Monitoring or laboratory testing	43	6.02 (1.54)	5.96 (1.56)	6.21 (1.63)	5.50 (0.71)
Pregnancy or lactation^a^	43	5.95 (1.23)	5.85 (1.23)	6.43 (0.94)	4.00 (1.41)
Stability and compatibility	11	5.82 (1.08)	N/A	5.78 (1.09)^b^	6.00 (1.41)

^a^Participants were asked to initiate search using Watson Assistant for these scenarios. One participant was unable to use Watson Assistant because of technical issues and used the general search function.

^b^A total of 5 pharmacist participants received a specialty script rather than the general pharmacy script; therefore, the counts by question category differ; 5 pharmacists completed a scenario in the disease category, and 9 completed a scenario in the stability and compatibility category.

^c^N/A: not applicable.

**Table 5:** Average satisfaction by question category and role.

Question category	Tasks, n	Overall average (SD)	Physicians, nurse practitioner, and physician’s assistants (n=27), average (SD)	Pharmacists (n=14), average (SD)	Registered nurses (n=2), average (SD)
Adverse drug reaction or toxicity^a^	43	6.19 (1.45)	5.89 (1.67)	6.71 (0.83)	6.50 (0.71)
Disease	32	5.38 (1.58)	5.33 (1.69)	5.60 (0.89)^b^	N/A^c^
Drugs of choice or indication or therapeutic	41	6.15 (1.42)	6.19 (1.21)	6.07 (1.82)	N/A
Dosing or kinetics	41	5.63 (1.80)	5.52 (1.95)	5.86 (1.51)	N/A
Drug administration	4	7.00 (3.13)	N/A	N/A	7.00 (0)
Interaction^a^	43	5.95 (1.84)	5.96 (1.85)	6.43 (1.28)	2.50 (2.12)
Monitoring or laboratory testing	43	5.98 (1.57)	5.81 (1.59)	6.36 (1.60)	5.50 (0.71)
Pregnancy or lactation^a^	43	6.16 (1.13)	6.04 (1.09)	6.43 (1.28)	6.00 (0)
Stability and compatibility	11	6.27 (1.27)	N/A	6.67 (0.71)^b^	4.50 (2.12)

^a^Participants were asked to initiate search using Watson Assistant for these scenarios. One participant was unable to use Watson Assistant because of technical issues and used the general search function.

^b^A total of 5 pharmacist participants received a specialty script rather than the general pharmacy script; therefore, the counts by question category differ; 5 pharmacists completed a scenario in the disease category, and 9 completed a scenario in the stability and compatibility category.

^c^N/A: not applicable.

**Table 6:** Average ease and average satisfaction by specialty script.

Specialty scripts			Role, n (%)		Average ease (SD)			Average satisfaction (SD)	
**Cardiology (n=5)**						6.11 (1.51)			6.06 (1.53)
	Pharmacist	3 (60)							
	Physician	2 (40)							
**Endocrinology (n=6)**						5.74 (1.71)			6.19 (1.47)
	Physician	5 (83)							
	Nurse practitioner	1 (17)							
**Hematology or oncology (n=5)**						5.71 (1.56)			6.11 (1.35)
	Pharmacist	1 (20)							
	Physician	4 (80)							
**Infectious diseases (n=5)**						4.89 (1.62)			5.37 (1.67)
	Pharmacist	1 (20)							
	Physician	4 (80)							
**Internal medicine (n=6)**						5.38 (1.71)			5.31 (1.88)
	Physician	6 (100)							
**Neurology (n=5)**						5.91 (1.44)			6.14 (1.33)
	Physician	5 (100)							
**Nursing (n=2)**						5.07 (1.69)			5.57 (1.79)
	Registered nurse	2 (100)							
**General pharmacy (n=9)**						6.02 (1.28)			6.44 (1.24)
	Pharmacist	9 (100)							

### Time on Task

The total average time to find an answer across all tasks was 1 minute, 57 seconds (SD 1 minute, 32 seconds; range 00 minutes, 15 seconds-11 minutes, 36 seconds). The pharmacists took longer to finish tasks for all the question categories. The greatest differences in average time between the pharmacists (4 minutes, 8 seconds, SD 4 minutes) and the physicians, NP, and PAs (1 minute, 33 seconds, SD 39 seconds) were in the disease question category and drugs of choice or indication or therapeutic category (pharmacists: 3 minutes, 17 seconds, SD 1 minute, 43 seconds; and physicians, NP, and PAs: 1 minute, 32 seconds, SD 54 seconds; Table 7).

**Table 7:** Time on task by question category and role.

Question category	Tasks, n	Average time (SD; range)			
		All participants	Physicians, nurse practitioner, and physician’s assistants (n=27)	Pharmacists (n=14)	Registered nurses (n=2)
Adverse drug reaction or toxicity^a^	43	01:35 (01:24; 00:15-06:04)	01:33 (01:28; 00:15-06:04)	01:38 (01:26; 00:32-05:15)	01:33 (00:34; 01:09-01:57)
Disease	32	01:57 (01:49; 00:29-11:11)	01:33 (00:39; 00:29-02:48)	04:08 (04:00; 01:46-11:11)^b^	N/A^c^
Drugs of choice or indication or therapeutic	41	02:08 (01:29; 00:22-06:42)	01:32 (00:54; 00:22-03:57)	03:17 (01:43; 00:39-06:42)	N/A
Dosing or kinetics	41	02:09 (01:34; 00:32-06:42)	01:59 (01:21; 00:32-05:29)	02:29 (01:56; 00:50-06:42)	N/A
Drug administration	4	01:49 (02:11; 00:30-05:05)	N/A	N/A	01:49 (02:11; 00:30-05:05
Interaction^a^	43	01:47 (01:16; 00:25-04:53)	01:29 (01:17; 00:25-04:53)	02:13 (01:04; 01:08-04:35)	02:38 (01:51; 01:20-03:57)
Monitoring or laboratory testing	43	01:45 (01:21; 00:24-06:21)	01:29 (01:30; 00:24-06:21)	02:08 (00:53; 00:59-03:46)	02:50 (00:52; 02:14-03:27)
Pregnancy or lactation^a^	43	02:12 (01:49; 00:20-11:36)	02:07 (02:06; 00:20-11:36)	02:16 (01:13; 00:30-05:10)	02:54 (02:00; 01:29-04:18)
Stability and compatibility	11	02:30 (01:23; 01:08-05:37)	N/A	02:24 (01:31; 01:08-05:37)^b^	02:59 (00:16; 02:47-03:10)

^a^Participants were asked to initiate search using Watson Assistant for these scenarios. One participant was unable to use Watson Assistant because of technical issues and used the general search function

^b^A total of 5 pharmacist participants received a specialty script rather than the general pharmacy script; therefore, the counts by question category differ; 5 pharmacists completed a scenario in the disease category, and 9 completed a scenario in the stability and compatibility category.

^c^N/A: not applicable.

### Search and Navigation Behavior

The pharmacists took more actions on average (5.14, SD 3.48) than the physicians, NP, and PAs (4.2, SD 3.44) to find the answer to their questions and used the Ctrl-F or *Find on Page* feature in 30% (29/98) of the scenarios versus 15.3% (29/189) of the scenarios for the physicians, NP, and PAs (Table 8). In addition, in 31% (25/81) of the scenarios completed by the physicians, NP, and PAs and in 36% (15/42) of the scenarios completed by the pharmacists, they switched from Watson Assistant to view content on the main pages to obtain additional detail or because they were unable to find a satisfactory answer in Watson Assistant. Multiple general search entries and multiple Watson Assistant entries per scenario occurred in 28.7% (31/108) and 27% (22/81) of the scenarios, respectively, for the physicians, NP, and PAs. The pharmacists entered multiple general search entries per scenario in 41% (23/56) of the tasks.

**Table 8:** Navigation and search behavior by role.

Navigation and search actions	Physicians, nurse practitioner, and physician’s assistants	Pharmacists	Registered nurses
Number of tasks using *Find on Page* or Ctrl-F, n (%)	29^a^ (15.3)	29^b^ (29.6)	0^c^ (0)
Count of switch from Watson Assistant to main content, n (%)	25^d^ (30.9)	15^e^ (35.7)	4^f^ (66.7)
Count of multiple general search entries, n (%)	31^g^ (28.7)	23^h^ (41.1)	3^i^ (37.5)
Count of multiple entries in Watson Assistant, n (%)	22^d^ (27.2)	7^e^ (16.7)	3^f^ (50)
Count of actions (clicks on search result, navigation menu, or entered text), average (SD)	4.2 (3.44)	5.14 (3.48)	5.92 (3.40)

^a^n=189.

^b^n=98.

^c^n=14.

^d^n=81.

^e^n=42.

^f^n=6.

^g^n=108.

^h^n=56.

^i^n=8.

### Posttest Interview Responses

Overall, 81% (35/43) of the participants felt that the information provided was accurate and reliable. Of the 14 pharmacists, 8 (57%) preferred the combined solution over their current system, and 10 (71%) would recommend the solution to their colleagues, whereas of the 27 physicians, NP, and PAs, 9 (33%) would prefer the combined solution, and 13 (48%) would recommend it to their colleagues (Table 9).

**Table 9:** Posttest interview responses by role and Micromedex experience (N=43).

Question and role			Yes, n (%)		No, n (%)		Maybe, n (%)		Not yet, n (%)		I don’t know, n (%)
**Would you recommend this tool to your colleagues?**											
	Physicians, nurse practitioner, and physician’s assistants (n=27)	13 (48)		8 (30)		1 (4)		2 (7)		3 (11)	
	Pharmacists (n=14)	10 (71)		1 (7)		1 (7)		2 (14)		0 (0)	
	Registered nurses (n=2)	2 (100)		0 (0)		0 (0)		0 (0)		0 (0)	
	All roles	25 (58)		9 (21)		2 (5)		4 (9)		3 (7)	
**Did you feel the information was accurate and reliable?**											
	Physicians, nurse practitioner, and physician’s assistants (n=27)	21 (78)		1 (4)		5 (19)		N/A^a^		N/A	
	Pharmacists (n=14)	12 (86)		0 (0)		2 (14)		N/A		N/A	
	Registered nurses (n=2)	2 (100)		0 (0)		0 (0)		N/A		N/A	
	All roles	35 (81)		1 (2)		7 (16)		N/A		N/A	
**Would you prefer using DynaMed and Micromedex with Watson?**											
	Physicians, nurse practitioner, and physician’s assistants (n=27)	9 (33)		11 (41)		3 (11)		4 (15)		N/A	
	Pharmacists (n=14)	8 (57)		4 (29)		1 (7)		1 (7)		N/A	
	Registered nurses (n=2)	2 (100)		0 (0)		0 (0)		0 (0)		N/A	
	All roles	19 (44)		15 (35)		4 (9)		5 (12)		N/A	

^a^N/A: not applicable.

### Participant Observations and Feedback

Think aloud and observations of user behavior highlighted usability issues with the combined tool. Participants experienced challenges with both the general search function and Watson Assistant in their ability to provide an exact match for their search. Participants often encountered issues when a search term was misspelled, an acronym or abbreviation was used, an unrecognized synonym was used, or too many words were entered. Another category of usability issues was related to the formatting and organization of the content pages. While looking through the content, participants made suggestions for additional features to help locate information on the page (more graphs, tables, embedded links, visual cues, and consistency in formatting). Other issues were specific to Watson Assistant, particularly related to the challenges that participants experienced with closing the dialog box and understanding the interaction with the *clear* button and search terms.

In response to the posttask interview questions, participants shared that they liked the integration of the condition-specific knowledge with the detailed drug information. They also liked the inclusion of guidelines and citations accessible through hyperlinks. In addition, participants liked the predictive text and contextual suggestions in the general search. The drug-drug interaction feature of Watson Assistant as well as Watson Assistant’s ability to prompt them in a way that would narrow in on an answer were frequently mentioned as something they liked.

Participants reported their reasons for feeling that the information was accurate and reliable. The reasons included the following: answers matched their prior knowledge or experience in clinical settings, the tool cited evidence-based research such as clinical trials and guidelines, the evidence did not seem to be influenced by drug companies, and the tool was comprehensive with in-depth answers.

Participants disliked that the general search function and Watson Assistant both required specific words or phrasing to return quick and relevant results. They also expressed their dislike of the dense text, describing the length of time required to find an answer. Participants reported feeling as though they were not able to find answers to questions requiring more subspecialty specific knowledge.

## Discussion

### Principal Findings

Web-based medical information resources with technologies to support search are common, but few studies have been conducted to assess or improve their usability or to evaluate the ability of such tools to answer questions. We evaluated user interactions with the DynaMed and Micromedex with Watson combined solution and identified strengths as well as potential opportunities for improvement. We found that there was considerable variability in the time spent on each task. One reason for this included participant differences in their approach to consuming information; some were more interested in looking at additional detail and references, whereas others were satisfied with a general answer. This behavior is consistent with a prior study where physicians reported that a barrier to using electronic resources was related to difficulty in knowing when to stop searching for an answer [6]. Time on task could also have been influenced by participants’ prior use of POCI tools because a connection has been reported between participants’ prior experience and time to answer as well as confidence and satisfaction with the answer found [4]. Thus, the level of prior experience with these types of tools may also explain some of the differences seen among participants in terms of task ease and satisfaction.

Overall, pharmacists found DynaMed and Micromedex with Watson easier to use than other provider groups and were more satisfied with the answers (Table 5). Pharmacists generally had more experience with Micromedex, which may account for some of these findings. Pharmacists who had more experience with Micromedex may have had the advantage of understanding how best to articulate clinical questions for entry into the general search function to obtain helpful answers. In addition, pharmacists spent more time on tasks and performed more actions to find their answers. This extra effort could reflect their clinical role, where pharmacists routinely use POCI tools to help answer drug information questions for other clinicians. In comparison, the physicians, NP, and PAs had a different experience in which they tended to finish tasks in <2 minutes, with average ease and satisfaction ratings lower than those of pharmacists. Internal medicine physicians were the least satisfied with their answers. Physicians have been known in practice to experience considerable time constraints and cognitive burden from computer use [24,25]. Issues observed and verbalized by physicians included challenges scanning the text and the organization and visual hierarchy of the content pages, as well as the desire to have more curated knowledge useful for clinical practice. These results indicate that different requirements may be necessary to meet the needs of clinical pharmacists compared with those of practicing physicians, NPs, and PAs. These factors may explain why more pharmacists would recommend the combined tool to their colleagues than the physicians, NP, and PAs. It could also explain why more pharmacists would prefer using the combined tool over what they currently use than the physicians, NP, and PAs.

However, some changes could make the combined solution more accessible to physicians and RNs. Search functions that support a wider variety of differences in clinicians’ formation of clinical questions for general searches would be advantageous. A research study showed that medical residents had difficulty creating “answerable questions” to produce a general search that would provide the most relevant evidence [6]. Watson Assistant would also benefit from being able to accommodate more natural language used by clinicians, such as abbreviations, acronyms, and synonyms. A previous study demonstrated that the Watson Assistant conversational agent was able to match the intent of 80% of the queries answerable by the conversational agent [7]. Our study was able to examine the user interaction of different clinical roles with the combined solution through a set of standard scenarios known to be answerable by DynaMed and Micromedex with Watson. We found that although participants were successful and often able to ultimately find an answer, more than half of the participants (24/42, 57%) had to enter search terms in Watson Assistant multiple times, and slightly more than two-thirds of the participants (29/42, 69%) viewed website content outside of Watson Assistant to obtain more information at least once. In addition, for many of the participants (28/43, 65%), particularly pharmacists, use of the Ctrl-F feature to find information on the content pages was key to their success. Research on the usability of these types of tools and observations of clinician use is sparse. We found only 1 closely related study focusing on a comparison of efficiency, satisfaction, and accuracy of 2 tools, DynaMed and UpToDate [4]. The study found that clinicians were more satisfied and found their answer more quickly with UpToDate, although the accuracy of the answers was similar using both tools. The authors hypothesized that greater familiarity with UpToDate may have influenced these results [4]. Our study aimed to uncover potential usability issues or use patterns by role that could help explain differences in satisfaction and efficiency in tools such as these. Studying the more detailed interactions with the system could have important benefits for practicing clinicians if the issues identified are shared with users and addressed in product enhancements.

### Limitations

This study includes several limitations. First, users may have reasoned that the usability issues they encountered were not related to the design or performance of the DynaMed and Micromedex with Watson combined tool but rather because of the users’ own unfamiliarity with the tool [26]. This explains situations where the users struggled to find the answer but still provided high ratings when responding to their posttask questions. Second, we conducted usability testing with clinicians who all practice at the same organization, which may affect generalization of our findings to other institutions using DynaMed and Micromedex with Watson. Clinicians’ information-seeking behaviors may differ according to a variety of factors such as age, experience with technology, medical experience, geographic location, and access to resources [27]. Testing a larger number of participants and additional subgroups of users might offer more validity for generalizing findings [28]. Thus, it is important to continue usability testing with a wide range of clinicians to ensure that all user experiences can be recognized and addressed.

Third, although we attempted to standardize scripts by clinical question category and type, the scripts still contained differences (eg, question difficulty and wording). These differences could have affected participants’ experiences in understanding and answering the questions. In addition, creating individual scripts for specific roles (RNs and pharmacists) as well as specific specialties resulted in some inconsistencies related to which script to use for specific participants, for example, specialty pharmacists were interviewed using a specialty script instead of the general pharmacy script. This was done to reach sufficient sample sizes for the specialties that were more difficult to recruit. However, this proved to be helpful in providing insight into whether outcomes were influenced by specialty. Fourth and last, our study was conducted during the COVID-19 pandemic resulting in challenges recruiting a larger number of participants for each specialty. This was especially true for interested RN participants whose availability was greatly reduced by the increased demand of resources needed for patient care. To address both safety and scheduling concerns, usability testing sessions were conducted remotely, which may have influenced the results in comparison with in-person testing [29]. Additional testing with clinicians in various specialties may provide meaningful insights into the types of questions relevant to their practice, interaction behavior, and workflow considerations for these specialties, which could help to identify and prioritize tailored improvements to the combined tool.

### Conclusions

This study is one of the first to test the usability of the DynaMed and Micromedex with Watson combined solution, now known as DynaMedex. It is also one of the first studies to compare ease and satisfaction of answers to questions in various content categories and by clinician role. We found that although the application performed well overall, pharmacists were able to use it most effectively in finding answers, whereas physicians and RNs had more difficulty finding the information they needed. We identified multiple changes that could be made to the tool to improve its usability, especially for inexperienced users. Understanding the determinants of information-seeking behavior is key to aiding physicians with finding answers to drug and disease management questions at the point of care.

## Appendix

Multimedia Appendix 1 Internal medicine usability test script: moderator guide.

## Abbreviations

AI: artificial intelligence
NLP: natural language processing
NP: nurse practitioner
PA: physician’s assistant
POCI: point-of-care information
RN: registered nurse
