# Correction: Performance of a Web-Based Reference Database With Natural Language Searching Capabilities: Usability Evaluation of DynaMed and Micromedex With Watson

**DOI:** 10.2196/48468

**Published:** 2023-05-18

**Authors:** Angela Rui, Pamela M Garabedian, Marlika Marceau, Ania Syrowatka, Lynn A Volk, Heba H Edrees, Diane L Seger, Mary G Amato, Jacob Cambre, Sevan Dulgarian, Lisa P Newmark, Karen C Nanji, Petra Schultz, Gretchen Purcell Jackson, Ronen Rozenblum, David W Bates

**Affiliations:** 1 Division of General Internal Medicine Brigham and Women's Hospital Boston, MA United States; 2 Clinical and Quality Analysis Mass General Brigham Somerville, MA United States; 3 Harvard Medical School Boston, MA United States; 4 Massachusetts College of Pharmacy and Health Sciences (MCPHS) Boston, MA United States; 5 Department of Anesthesia Critical Care and Pain Medicine Massachusetts General Hospital Boston, MA United States; 6 Merative Cambridge, MA United States; 7 Vanderbilt University Medical Center Nashville, TN United States; 8 Intuitive Surgical Sunnyvale, CA United States; 9 Harvard TH Chan School of Public Health Boston, MA United States

In “Performance of a Web-Based Reference Database With Natural Language Searching Capabilities: Usability Evaluation of DynaMed and Micromedex With Watson” (JMIR Res Protoc 2023;25:e47678) the authors noted two errors.

1. The Authors Contributions section currently reads as:

All authors contributed to the study conception; design; and acquisition, analysis, or interpretation of the data. AR, PMG, AS, LAV, DLS, GPJ, and DWB were responsible for study conception or design. PMG, HHE, DLS, and MGA developed the interview guides. MM, AS, SD, and LPN conducted participant recruitment. PMG acted as the interview moderator and had either AR or MM assisting with data collection during testing. PMG, MM, JC, and SD abstracted the data from interview recordings. Data analysis was performed by PMG, MM, and AR. The first draft of the manuscript was written by AR and PMG, with all authors reviewing the draft and providing critical feedback. All authors contributed to and approved the final manuscript.

And will be changed to:

*RR and DWB are co-senior authors and contributed equally.*

All authors contributed to the study conception; design; and acquisition, analysis, or interpretation of the data. AR, PMG, AS, LAV, DLS, GPJ, and DWB were responsible for study conception or design. PMG, HHE, DLS, and MGA developed the interview guides. MM, AS, SD, and LPN conducted participant recruitment. PMG acted as the interview moderator and had either AR or MM assisting with data collection during testing. PMG, MM, JC, and SD abstracted the data from interview recordings. Data analysis was performed by PMG, MM, and AR. The first draft of the manuscript was written by AR and PMG, with all authors reviewing the draft and providing critical feedback. All authors contributed to and approved the final manuscript.

2. In the original article, the ORCID number for Petra Schultz was reported as follows:

0000-0001-7337-1046

And has been updated to:

0000-0001-7949-9243

The correction will appear in the online version of the paper on the JMIR Publications website on May 18, 2023, together with the publication of this correction notice. Because this was made after submission to PubMed, PubMed Central, and other full-text repositories, the corrected article has also been resubmitted to those repositories.

