# Supplementary material for: Performance of a Web-Based Reference Database With Natural Language Searching Capabilities: Usability Evaluation of DynaMed and Micromedex With Watson
Source: JMIR Hum Factors. 2023 Apr 17;10:e43960. doi: 10.2196/43960 (PMC10152386; doi:10.2196/43960)
Supplement: Multimedia Appendix 1 [file humanfactors_v10i1e43960_app1.docx]

**Appendix A**

**Internal Medicine Usability Test Script: Moderator Guide**

**INTRODUCTION**

Thank you for participating today. You will be helping us test a clinical decision support solution for answering questions about medications and /or diseases. You will be looking at a stand-alone web version of the tool not integrated into Epic for this testing.

During the session, we will do a brief introduction to the site and give you a minute to explore. Then, we will provide you with a few clinical scenarios where we’ll ask you to use the site to answer some questions. We’ll also ask you to use the site to answer a few of your own questions based on recent experience. We will then talk briefly about your experience using the Dynamed and Micromedex with Watson tool, called Dynamedex. Please speak aloud as you are using the tool (talk about what you’re thinking, what you’re expecting to happen when you click on certain things, your reactions to elements in the tool). If you are silent for a while, I may remind you to keep talking aloud.

My role is that of a neutral observer. I will not be able to answer questions about how to use the tool during the usability test, but we can review any questions at the end.

We will be recording our Zoom session together. You’ll be sharing your screen while you use the site. The video and audio will be seen by only those people working on the research project and will be used in our analysis of the tool.

Do you have any questions? Is it okay with you to record?

# **Demographic Questions**

I’d like to ask you a couple of questions before we start.

1. What is your clinical role?
2. How many years have you been practicing?
3. Do you primarily work in the inpatient or outpatient setting?
4. Do you work at Brigham and Women’s Hospital (or have an appointment at BWH)?
5. What reference database do you use to look up information regarding medications and /or diseases currently?
6. How often do you use Micromedex? Ask Watson? Dynamed?
   1. Never
   2. A few times a year
   3. Once a month
   4. Once a week
   5. Daily

# **SCENARIOS**

During this portion of the test, you will read a scenario aloud. You’ll then use the site to address the task in the scenario. Each task will direct you to use either the search bar or the Ask Watson feature. [Open site]. One bug that want to make you aware of, is that when you use the search feature the list of results you see may sometimes bring you to the bottom of the page and you’ll need to scroll up. After each task, I’ll ask you a couple of questions about how it went.

*Scenario 1: A 19-year-old female with bulimia presents and is suspected to have body dysmorphic disorder. What is the clinical presentation and diagnostic criteria for body dysmorphic disorder? (Use search bar)*

Post Task Questions:

Overall, how easy/difficult was it to find an answer? (1=Very Difficult, 7 Very Easy)

1  2  3  4  5  6  7

Overall, how satisfied were you with the answer? (1=Very Dissatisfied, 7=Very Satisfied)

1  2  3  4  5  6  7

*Scenario 2: A mother with a 2-week-old infant she is breast feeding asks you if it is okay for her to take hydroxychloroquine while breastfeeding her newborn. What do you recommend? (Use Ask Watson)*

Post Task Questions:

Overall, how easy/difficult was it to find an answer? (1=Very Difficult, 7 Very Easy)

1  2  3  4  5  6  7

Overall, how satisfied were you with the answer? (1=Very Dissatisfied, 7=Very Satisfied)

1  2  3  4  5  6  7

*Scenario 3: You have an obese inpatient who requires primary VTE prophylaxis while in the hospital. What dose of subcutaneous unfractionated heparin should be used for this indication? (Use search bar)*

Post Task Questions:

Overall, how easy/difficult was it to find an answer? (1=Very Difficult, 7 Very Easy)

1  2  3  4  5  6  7

Overall, how satisfied were you with the answer? (1=Very Dissatisfied, 7=Very Satisfied)

1  2  3  4  5  6  7

*Scenario 4: You started your patient on Singulair. What lab values should be monitored? (Use search bar)*

Post Task Questions:

Overall, how easy/difficult was it to find an answer? (1=Very Difficult, 7 Very Easy)

1  2  3  4  5  6  7

Overall, how satisfied were you with the answer? (1=Very Dissatisfied, 7=Very Satisfied)

1  2  3  4  5  6  7

*Scenario 5: A patient reports experiencing excessive sweating and is wondering if the medication (venlafaxine) she is taking is causing this side effect. Is it possible for venlafaxine to cause excessive sweating? (Use Ask Watson).*

Post Task Questions:

Overall, how easy/difficult was it to find an answer? (1=Very Difficult, 7 Very Easy)

1  2  3  4  5  6  7

Overall, how satisfied were you with the answer? (1=Very Dissatisfied, 7=Very Satisfied)

1  2  3  4  5  6  7

*Scenario 6:* *A patient with rheumatoid arthritis receiving methotrexate (MTX) will be receiving the influenza vaccine (live flu vaccine - Flumist intranasal). Is there an interaction between MTX and this influenza vaccine? (Use Ask Watson)*

Post Task Questions:

Overall, how easy/difficult was it to find an answer? (1=Very Difficult, 7 Very Easy)

1  2  3  4  5  6  7

Overall, how satisfied were you with the answer? (1=Very Dissatisfied, 7=Very Satisfied)

1  2  3  4  5  6  7

*Scenario 7:* *A patient being treated with azathioprine (Imuran) for Crohn's disease is identified to have thiopurine methyltransferase (TPMT) deficiency. What modifications to their azathioprine therapy should be made? (Use search bar)*

Post Task Questions:

Overall, how easy/difficult was it to find an answer? (1=Very Difficult, 7 Very Easy)

1  2  3  4  5  6  7

Overall, how satisfied were you with the answer? (1=Very Dissatisfied, 7=Very Satisfied)

1  2  3  4  5  6  7

# **Clinician’s Own Questions for Testing**

Please try answering your own question using the tool based on your recent experience. You can use either the search bar or Ask Watson.

1. Clinician’s question:

Post Task Questions:

Overall, how easy/difficult was it to find an answer? (1=Very Difficult, 7 Very Easy)

1  2  3  4  5  6  7

Overall, how satisfied were you with the answer? (1=Very Dissatisfied, 7=Very Satisfied)

1  2  3  4  5  6  7

1. Clinician’s question:

Post Task Questions:

Overall, how easy/difficult was it to find an answer? (1=Very Difficult, 7 Very Easy)

1  2  3  4  5  6  7

Overall, how satisfied were you with the answer? (1=Very Dissatisfied, 7=Very Satisfied)

1  2  3  4  5  6  7

1. Clinician’s question:

Post Task Questions:

Overall, how easy/difficult was it to find an answer? (1=Very Difficult, 7 Very Easy)

1  2  3  4  5  6  7

Overall, how satisfied were you with the answer? (1=Very Dissatisfied, 7=Very Satisfied)

1  2  3  4  5  6  7

# **Semi-Structured Post-Test Interview**

We have a few final questions to ask you about the tool.

1. What did you like about this tool?
2. What did you dislike about this tool?
3. What else do you feel this tool should do?
4. Would you recommend this tool to your colleagues? Why?
5. Did you feel the information you found was accurate and reliable? Why or why not?
6. Would you prefer using DynaMed and Micromedex with Watson over what you currently use to find information on diseases and drug therapy? Why?
7. Anything else you would like to share with us about the tool or your experience today?
